# RAP Tag and PMab-2 Antibody: A Tagging System for Detecting and Purifying Proteins in Plant Cells

**DOI:** 10.3389/fpls.2020.510444

**Published:** 2020-09-10

**Authors:** Kenji Miura, Hideki Yoshida, Shohei Nosaki, Mika K. Kaneko, Yukinari Kato

**Affiliations:** ^1^ Faculty of Life and Environmental Sciences, University of Tsukuba, Tsukuba, Japan; ^2^ Tsukuba-Plant Innovation Research Center, University of Tsukuba, Tsukuba, Japan; ^3^ Department of Antibody Drug Development, Tohoku University Graduate School of Medicine, Sendai, Japan; ^4^ New Industry Creation Hatchery Center, Tohoku University, Sendai, Japan

**Keywords:** transient expression, tagging system, RAP tag, agroinfiltration, monoclonal antibody, protein expression, protein purification, plant biochemistry

## Abstract

An affinity tag system requires both high affinity and specificity. The RAP tag epitope DMVNPGLEDRIE, derived from rat podoplanin (PDPN), is specifically recognized by PMab-2 monoclonal antibodies in rats. Here, we demonstrated that high levels of PMab-2 can be produced in *Nicotiana benthamiana* and plant-derived PMab-2 possesses similar activity to CHO-derived PMab-2, and the RAP tag presents a useful tagging system for detecting and purifying proteins from plant cells. The heavy chain of PMab-2 fused with KDEL, an endoplasmic reticulum retention sequence, and the light chain of the antibody were introduced into *N. benthamiana* by agroinfiltration. The expression of PMab-2 peaked 4 days after agroinfiltration, and approximately 0.3 mg/g fresh weight of the antibody was accumulated. After purification, the plant-derived PMab-2 successfully recognized rat PDPN expressed in CHO-K1 cells and exhibited almost the same binding activity as CHO-derived PMab-2. The RAP-tagged proteins expressed in plant cells were specifically recognized by PMab-2. These results indicate that PMab-2 can accumulate at high levels in *N. benthamiana* and is easily purified and that the RAP tagging system presents a useful tool for detecting and purifying proteins of interest in plant cells.

## Introduction

Affinity tag systems are useful for detecting and purifying target proteins and are classified into peptide and protein tags. Protein tags, including green fluorescent protein (GFP), *β-*glucuronidase (GUS), and HaloTag (Lang et al., 2006), are used in plant cells and are generally large in size. Although these tags are very useful as reporter proteins to detect protein localization or expression levels (Miura et al., 2013; Ohta et al., 2018), they sometimes affect the characteristics of the target proteins. Peptide tags, such as the FLAG and HA tags, are also commonly used in plant cells. These small tags are less likely to affect the structure and function of target proteins (Book et al., 2010). However, these peptide tags are sometimes not suitable, because of their low specificity, low affinity, or difficulty in achieving protein elution. For example, if the expression level of the His-tagged target protein is low in the plant cells, the antibodies cannot detect the target proteins easily due to cross-reactions with other metal-binding proteins (Lichty et al., 2005). To assess different target proteins in plant cells, various tags are required. Immunoaffinity chromatography, which uses anti-peptide monoclonal antibodies (mAbs), is a very powerful tool for purifying scarce recombinant proteins, because of its high specificity and affinity.

Previously, a mouse mAb (clone PMab-2) was used against the platelet aggregation-stimulating domain of rat podoplanin (PDPN) (Oki et al., 2015). The PMab-2 antibody has high specificity for rat PDPN; however, it does not react with human and mouse PDPN. This antibody possesses high affinity and specificity for the RAP epitope tag DMVNPGLEDRIE, which consists of 12 amino acids (Fujii et al., 2017). On expressing human epidermal growth factor receptor (EGFR) fused with RAP tag in CHO-K1 and LN229 cells, a single, strong band of EGFR-RAP was detected by PMab-2 (Fujii et al., 2017). These results suggest that the PMab-2 and RAP system can be used in plant cells. If a high level of antibody production is achieved in plant cells, the cost of antibody production may be reduced.

Because plants can generate large quantities of proteins at low cost and present low risk of contamination by animal or human pathogens, they have been proposed as bioreactors for producing biosimilar recombinant proteins (Tschofen et al., 2016; Donini and Marusic, 2019). Recombinant proteins are produced in plants using two different systems, i.e., stable genetic transformation and transient gene expression (Donini and Marusic, 2019). The production of recombinant proteins by transgenic plants is highly time-consuming and often results in a low yield (Tschofen et al., 2016). In contrast, a high yield of recombinant proteins can be obtained within 1–2 weeks by a transient gene expression system with a “deconstructed” viral vector system (Ibrahim et al., 2019). The magnICON system, based on the tobacco mosaic virus replication system, is one of the most renowned deconstructed viral vector systems (Gleba et al., 2005; Marillonnet et al., 2005). We recently developed one of the most efficient transient protein expression systems in plant cells, called the Tsukuba system (Yamamoto et al., 2018; Hoshikawa et al., 2019; Suzaki et al., 2019). The pBYR2HS vector exhibits geminiviral replication and includes a double terminator. On expressing GFP in *Nicotiana benthamiana* using this system, approximately 4 mg/g fresh weight (FW) of GFP was able to be produced (Yamamoto et al., 2018).

To determine if a protein complex can also by produced by the Tsukuba system, PMab-2 was expressed in *N. benthamiana* in the present study. The heavy chain (HC) of PMab-2 was fused with KDEL, an endoplasmic reticulum (ER) retention sequence, and was co-expressed with the light chain (LC) of PMab-2, resulting in the formation of an immune complex. The plant-produced PMab-2 exhibited similar activity to that produced in CHO cells. Furthermore, RAP-tagged target proteins were expressed in plant cells, and then detected specifically and purified using the PMab-2 antibody. The results indicated that the RAP tag can be used for both protein detection in plant cells using western blotting and protein purification from plant soluble extracts.

## Materials and Methods

### Vector Construction and Preparation of the *Agrobacterium* Suspension

*N. benthamiana* codon-optimized HC, fused with SEKEDL at the C-terminus for retention at the ER, and LC genes of the PMab-2 monoclonal antibody (Fujii et al., 2017) were synthesized using GeneArt Strings DNA Fragments (Thermo Fisher Scientific). The HC and LC genes were amplified using the primers pBYR2HS-MYL-F and pBYR2HS-KDEL-R, and pBYR2HS-MRF-F and pBYR2HS-stopC-R (**Supplemental Table S1**), respectively. The PCR products were introduced into *Sal*I-digested pBYR2HS (Yamamoto et al., 2018), using the In-Fusion HD Cloning Kit (Takara Bio). The resulting constructs were designated as either pBYR2HS-PMab2H or pBYR2HS-PMab2L, respectively.

*Agrobacterium tumefaciens* GV3101, harboring either pBYR2HS-PMab2H or pBYR2HS-PMab2L, were grown separately in L-broth medium containing 10 mM MES (pH 5.6), 20 µM acetosyringone, 100 mg/L kanamycin, 30 mg/L gentamycin, and 30 mg/L rifampicin, up to the stationary phase, at 28°C. The *Agrobacterium* culture was then centrifuged at 3,700 ×*g* for 15 min, the supernatant was discarded, and the *A. tumefaciens* pellet was resuspended in the infiltration buffer [10 mM MgCl_2_, 10 mM MES (pH 5.6), and 100 µM acetosyringone]. The concentration of *A. tumefaciens* in the suspension was adjusted to OD_600_ = 1. The suspensions of *A. tumefaciens* harboring pBYR2HS-PMab2H and pBYR2HS-PMab2L were mixed at a ratio of 1:1.

### Plant Growth Conditions and Agroinfiltration

*N. benthamiana* plants were grown for 5–6 weeks, at 25°C and under a 16-h light/8-h dark photoperiod. Five hundred milliliters of the mixed *A. tumefaciens* suspension was poured into a beaker. Plant leaves were soaked in the suspension, and vacuum infiltration was performed (Leuzinger et al., 2013). After infiltration, *N. benthamiana* were incubated at 20°C in a growth chamber, under a 16-h light/8-h dark photoperiod.

### Purification of Recombinant PMab-2 Antibodies From Agroinfiltrated Leaves

PMab-2 was purified following a previously described method (Ocampo et al., 2016) with some modifications. Briefly, 10 g of agroinfiltrated *N. benthamiana* leaves were ground in liquid nitrogen using a mortar and pestle. The powdered tissue was mixed with 40 ml of extraction buffer (0.5 NaCl, 45 mM Tris-HCl, 1 mM EDTA, 40 mM ascorbic acid, 1 mM PMSF; pH 7.5) and then agitated on ice for 1 h. The suspension was filtered through Miracloth (Meck Millipore), and centrifuged twice at 44,000 ×*g* for 30 min at 4°C. The supernatant was filtered through an Omnipore Membrane Filter (pore size 0.2 µm; Merck Millipore). PMab-2 was then purified using the AKTA start system equipped with a HiTrap Protein G HP column (GE Healthcare). Once the antibodies had bound to Protein G, the column was washed with the aforementioned extraction buffer without ascorbic acid. The antibodies were eluted with 0.1 M glycine-HCl (pH 2.7) and neutralized with 60 µl of 1 M Tris-HCl (pH 9.0) per ml fraction. The elutant was concentrated using Vivaspin (Sartorius) with 1 × PBS, at approximately 20 times.

### Construction of RAP Tag Fused With Phytochrome Interacting Factor 4 (PIF4), the C-Terminal Region of BIG, and Bet v 1

To introduce the RAP tag into the pBYR2HS vector, PCR products containing the His-tag (HHHHHH), RAP tag (DMVNPGLEDRIE), and recognition site for HRV 3C protease (LEVLFQGP), were produced using the primers pBYR2HS-Hisx6-F, His-RAP-HRV3C, and pBYR2HS-HRV3C-R, or pBYR2HS-HRV3C-F, HRV3C-RAP-His, and pBYR2HS-stopHis-R, respectively (**Supplemental Table S1**). These two PCR products were then introduced into *Sal*I-digested pBYR2HS, using the In-Fusion HD Cloning Kit. The resulting constructs were designated as either pBYR2HS-NHisRAP or pBYR2HS-CRAPHis, respectively. The *SalI* recognition site in these vectors functioned as a cloning site.

Total RNA was extracted from *Arabidopsis thaliana* as previously described (Ohta et al., 2018). Briefly, total cDNA was synthesized using an oligo(dT) primer and SuperScript III Reverse Transcriptase (Thermo Fisher Scientific), according to the manufacturer’s instructions. *PIF4* was amplified using the primers pBYR2HS-dNPIF4-F and pBYR2HS-dNPIF4RH-R (**Supplemental Table S1**). The PCR products were introduced into *Sal*I-digested pBYR2HS-CRAPHis, using the In-Fusion HD Cloning Kit, and the resulting construct was designated as pBYR2HS-dNPIF4RH.

Specific cDNA for the *BIG* gene was synthesized from the total RNA of *A. thaliana*, using the BIG-15297R primer (**Supplemental Table S1**) and SuperScript III Reverse Transcriptase. This *BIG* cDNA was then used as a template to amplify the C-terminal region of *Arabidopsis* BIG gene, using the primers pBYR2HS-HR-BIG12466-F and pRI201-AtBIG-R (**Supplemental Table S1**). The PCR products were then introduced into *Sal*I-digested pBYR2HS-NHisRAP, using the In-Fusion HD Cloning Kit, and the resulting construct was designated as pBYR2HS-HRBIG12466.

*Nicotiana tabacum* codon-optimized *Bet v 1A* gene (Yamada et al., 2020) was amplified using the primers HRV3C-Betv1Nt-F and pRITetI-Betv1Nt-R (**Supplemental Table S1**). The PCR products were introduced into *Sal*I-digested pBYR2HS-NHisRAP, using the In-Fusion HD Cloning Kit, and the resulting construct was designated as pBYR2HS-HRBetv1Nt.

### Protein Extraction and Immunoblot Analysis

The soluble protein was prepared following a previously described protocol (Miura et al., 2012; Yamamoto et al., 2018). Briefly, plant leaves (100–200 mg) were ground, and lysis buffer [50 mM Tris-HCl, 120 mM NaCl, 0.2 mM sodium orthovanadate, 100 mM NaF, 10% glycerol, 0.2% Triton X-100, 5 mM DTT, and 1× protein inhibitor cocktail (Nacalai Tesque, Inc., Kyoto, Japan); pH 8.0] was added to the powdered leaves to obtain a concentration of 0.2 mg FW/μl. The mixture was incubated for 1 h. The liquid solution obtained after removing debris by centrifugation served as the soluble protein extract. The solution was the diluted 10 folds using the lysis buffer (0.02 mg FW/μl). To load a crude extract equivalent to 0.1 mg FW, 5 μl of the sample solution was applied onto an SDS-PAGE gel, which was then stained with Coomassie Brilliant Blue (CBB). The protein was transferred onto a PVDF membrane (Amersham Hybond P PVDF, GE Healthcare). The blot was probed with plant-derived PMab-2 and detected using Luminata Forte Western HRP substrate (Millipore).

To compare the protein expression levels, all proteins were applied onto the same gel. The band intensity was measured using ImageJ software, and the protein concentration was calculated as described previously (Yamada et al., 2020). As shown in **Figure 3**, the indicated weight of the purified PMab2 antibody was loaded onto the gel. The standard line was calculated based on the band intensities and protein concentration. Protein weight was calculated using the standard line and then divided by 0.1 mg FW.

### Purification of RAP-Tagged and FLAG-Tagged Target Proteins

PIF4-RAP was expressed in *N. benthamiana* by infiltration with pBYR2HS-dNPIF4RH. Soluble extracts were prepared from *N. benthamiana* leaves. Plant-derived PMab-2, purified from the *N. benthamiana* leaves, was incubated with 50 μl of Dynabeads Protein G (Thermo Fisher Scientific). The Dynabeads Protein G-PMab-2 complex was then rinsed off with PBS, and the soluble extracts containing the PIF4-RAP protein were added to the complex and incubated. After washing the complex with PBS, the RAP-tagged proteins were extracted using 50 μl of elution buffer (50 mM glycine; pH 2.7) and neutralized with 3 μl of 1 M Tris-HCl (pH 9.0).

His-Flag-Bet v 1 or His-RAP-Bet v 1 was expressed in *N. benthamiana* by infiltration with pBYR2HS-HFBetv1Nt (Yamada et al., 2020) or pBYR2HS-HRBetv1Nt, respectively. After incubation for 4 days, plant leaves (5 g) were ground, the lysis buffer was added to the powdered leaves to obtain a concentration of 0.5 g FW/ml, and the soluble extract was prepared.

Dynabeads Protein G (50 μl; Thermo Fisher Scientific) was added to a magnetic stand to remove the supernatant. Then, 1 μg of the anti-FLAG antibody (anti-DYKDDDDK antibody, Fujifilm Wako Pure Chemical Corp.) or plant-derived PMab-2 was added. The Dynabeads Protein G-PMab-2 complex was then rinsed off with PBS, and the soluble extracts (5 ml) were added to the complex and incubated for 1 h. After washing the complex with PBS, the RAP- or FLAG-tagged proteins were extracted using an SDS buffer (60 mM Tris-HCl, 2% SDS, 10% glycerol, 5% 2-mercaptoethanol, and 0.025% bromophenol blue; pH 6.8). The extract was separated by SDS-PAGE, and western blot analysis was performed using anti-poly-His tag antibody (Abcam).

### Flow Cytometry

Cells were harvested by brief exposure to 0.25% trypsin/1 mM EDTA (Nacalai Tesque, Inc.). After rinsing with 0.1% BSA/PBS, the cells were treated with primary mAbs for 30 min at 4°C, and then with Alexa Fluor 488-conjugated anti-mouse IgG (1:1,000; Cell Signaling Technology, Inc., Danvers, MA). Fluorescence data were collected using EC800 Cell Analyzer (Sony Corp.).

## Results

### Transient Expression of Recombinant PMab-2 in *N. benthamiana* Leaves

**Figure 1:**
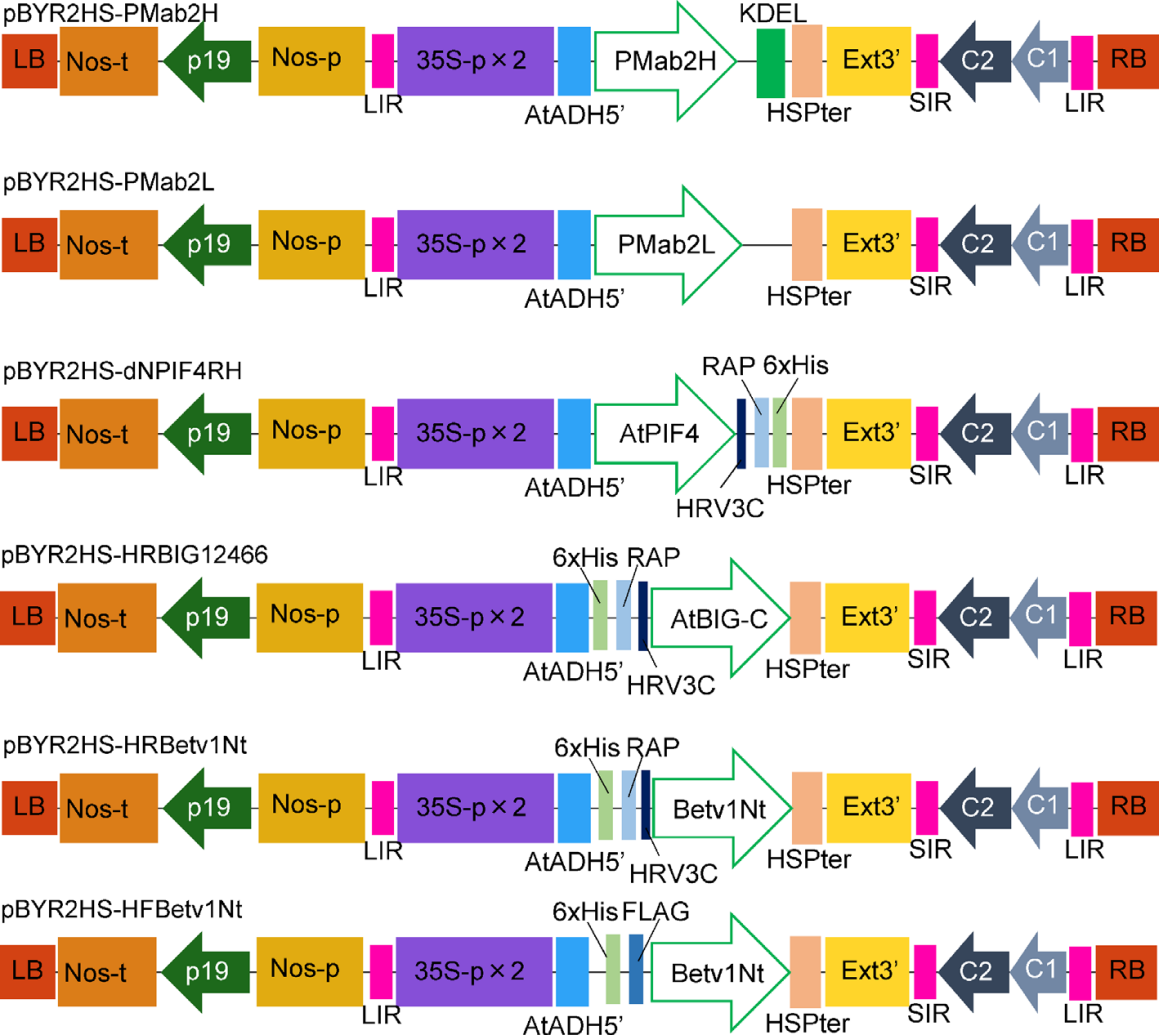
Schematic representation of the T-DNA regions of the plasmids, including pBYR2HS-PMab2H and pBYR2HS-PMab2L, used for protein expression. 35S-p × 2, CaMV 35S promoter with double-enhanced element; AtADH5′, 5′-untranslated region (UTR) of *Arabidopsis thaliana* alcohol dehydrogenase gene; KDEL, Lys-Asp-Glu-Leu sequence for endoplasmic reticulum retention; HSPter, heat shock protein gene terminator; Ext3′, tobacco extensin gene 3′ element; LIR, long intergenic region of the bean yellow dwarf virus (BeYDV) genome; SIR, short intergenic region of the BeYDV genome; C1/C2, BeYDV ORFs C1 and C2 encoding replication initiation protein (Rep) and RepA, respectively; LB and RB, left and right borders of the T-DNA region, respectively; Nos-p and Nos-t, NOS promoter and terminator, respectively; p19, a gene-silencing suppressor gene from tomato bushy stunt virus; HRV3C, HRV 3C protease recognition site.

Codon-optimized PMab-2 HC and LC were introduced into pBYR2HS to obtain pBYR2HS-PMab2H and pBYR2HS-PMab2L, respectively (**Figure 1**). A previous study demonstrated that the expression level of IgG in *N. benthamiana* leaves was higher when the HC of the antibody was expressed in the ER (Phoolcharoen et al., 2011). Thus, PMab-2 HC was fused with KDEL, an ER retention sequence. *Agrobacterium* harboring either pBYR2HS-PMab2H or pBYR2HS-PMab2L were incubated separately overnight. The two *Agrobacterium* solutions were then mixed and infiltrated into *N. benthamiana*.

**Figure 2:**
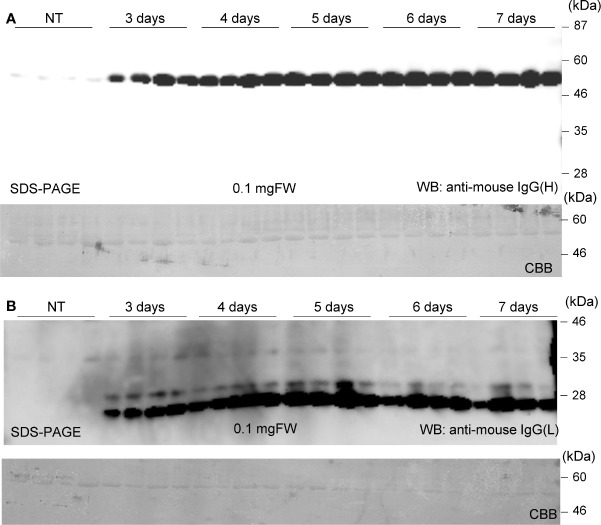
Expression of PMab-2 in *N. benthamiana* leaves. *N. benthamiana* leaves were infiltrated with an *Agrobacterium* mixture containing pBYR2HS-PMab2H and pBYR2HS-PMab2L and harvested at the indicated days post infiltration. Total protein extracts of *N. benthamiana* leaves were separated by 12% SDS-PAGE and transferred onto PVDF membranes. The membranes were incubated with anti-mouse IgG (H) to detect the HC **(A)** or anti-mouse IgG (L) to detect the LC **(B)**. Four samples each for HC and LC were subjected to SDS-PAGE. The large subunit of rubisco was detected by CBB staining as a loading control.

**Figure 3:**
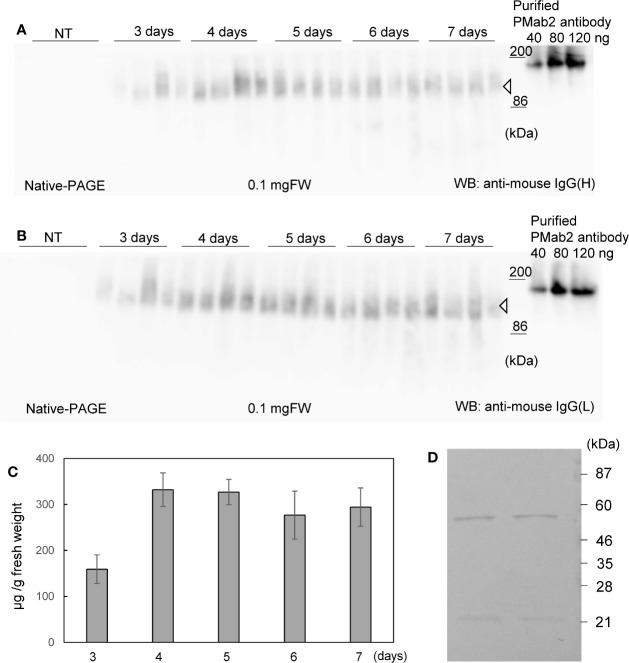
Expression and purification of PMab-2. *N. benthamiana* leaves were infiltrated with an *Agrobacterium* mixture containing pBYR2HS-PMab2H and pBYR2HS-PMab2L and harvested at the indicated days post infiltration. Total protein extracts of *N. benthamiana* leaves were separated by native-PAGE and transferred onto PVDF membranes. The membranes were incubated with anti-mouse IgG (H) to detect the HC **(A)** or anti-mouse IgG (L) to detect the LC **(B)**. Because these proteins formed a complex, bands of similar size were detected. Four samples each for HC and LC were loaded onto SDS-PAGE. **(C)** Protein concentration was measured based on the western blot band intensity using ImageJ software. **(D)** Purification of PMab-2 from protein extracts of *N. benthamiana* leaves infiltrated with pBYR2HS-PMab2H and pBYR2HS-PMab2L using the Protein G column. Purified PMab-2 were separated by SDS-PAGE and the gel was stained with CBB. Two representatives of four independent experiments are shown.

Soluble protein extracts were prepared from *N. benthamiana* leaves 3 to 7 days post infiltration. PMab-2 HC and LC were detected using anti-mouse IgG (H) antibody (**Figure 2A**) and anti-mouse IgG (L) antibody (**Figure 2B**), respectively. Western blot analyses revealed that PMab-2 HC and LC were expressed in *N. benthamiana* leaves, exhibiting the expected molecular weights of 50 and 25 kDa, respectively (**Figure 2**). The full tetrameric assembly of PMab-2 (2 HCs and 2 LCs) was confirmed by immunoblot analysis using native-PAGE (**Figures 3A, B**). Accumulation of PMab-2 was the highest 4 days post infiltration, exhibiting a value of approximately 0.3 mg/gFW (**Figure 3C**).

*N. benthamiana* expressing PMab-2 HC and LC were purified using a Protein G affinity column, as previously described (Husk et al., 2014). SDS-PAGE and CBB staining indicated that PMab-2 produced in *N. benthamiana* can be purified by a single affinity chromatography step, with a high level of purity and intact HC and LC (**Figure 3D**), indicating that the current purification method of PMab-2 worked well.

### Comparison of RAP Tag Recognition by Plant- and CHO Cell-Derived PMab-2

The RAP epitope tag sequence, DMVNPGLEDRIE, is included in PDPN (Fujii et al., 2017). PDPN was expressed in CHO-K1 cells, and recognition of the expressed PDPN by plant-derived PMab-2 was examined.

**Figure 4:**
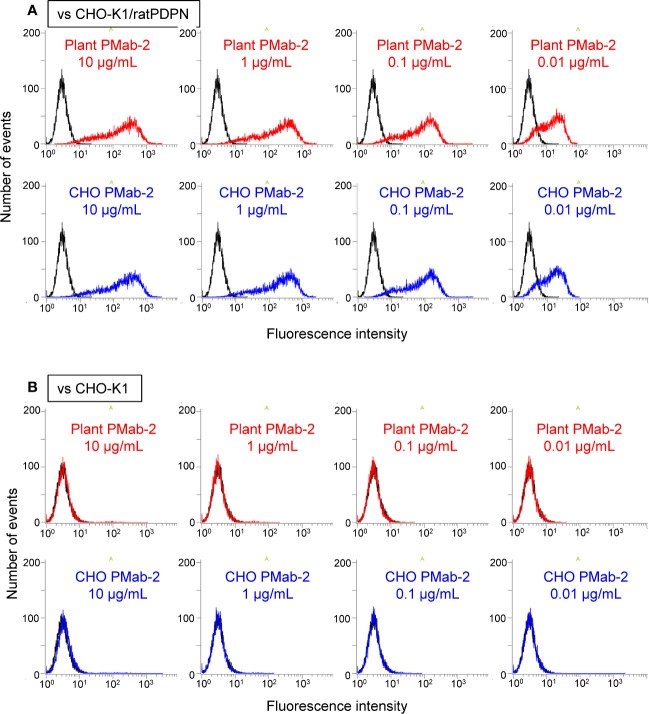
Fluorescence-activated cell sorting (FACS) analysis of rat podoplanin (PDPN) expressed in CHO-K1 cells. PMab-2 derived from *N. benthamiana* leaves or CHO cells was used. CHO-K1/rat PDPN cells **(A)** and CHO-K1 cells **(B)** were treated with the indicated concentration of PMab-2 purified from plant leaves or CHO cells, for 30 min at 4°C, followed by 1:1,000 dilution with Oregon Green 488 goat anti-mouse IgG. Fluorescence data were collected using Cell Analyzer EC800. Because it has previously been shown that CHO-derived PMab-2 can recognize the RAP tag in PDPN (Fujii et al., 2017), detection of PDPN from CHO-K1/rat PDPN cells by CHO-derived PMab-2 was established as the positive control non-detection of any protein from CHO-K1 cells by CHO-derived PMab-2 was established as the negative control.

Fluorescence-activated cell sorting (FACS) analysis indicated that plant-derived PMab-2 reacted with rat PDPN expressed in CHO-K1 cells in a manner similar to CHO-derived PMab-2 (**Figure 4A**). No significant differences were observed between CHO-K1 cells treated with plant-derived and CHO-produced PMab-2 (**Figure 4B**).

### The RAP Tag Is a Useful Peptide Tag for Plant Cells

PMab-2 shows high affinity and specificity for detecting RAP-tagged target proteins in mammalian cells (Fujii et al., 2017). To confirm the applicability of this affinity tag system in plant cells, RAP-tagged proteins were expressed and detected in plant cells using plant-derived PMab-2. *Arabidopsis* PIF4 is a basic helix-loop-helix-type transcription factor that interacts with the red light receptor phytochromes (Huq and Quail, 2002). The *Arabidopsis* BIG protein is a large, calossin-like protein required for polar auxin transport (Gil et al., 2001). The coding sequence of the *BIG* gene is approximately 15 kb long, hence the name “*BIG*”. In this study, the C-terminal region of *BIG* (2,769 bp) was used. It is difficult to produce PIF4 in an *E. coli* expression system. The BIG protein has not been analyzed extensively because of its large size. Thus, we determined if these proteins could be produced using our system.

**Figure 5:**
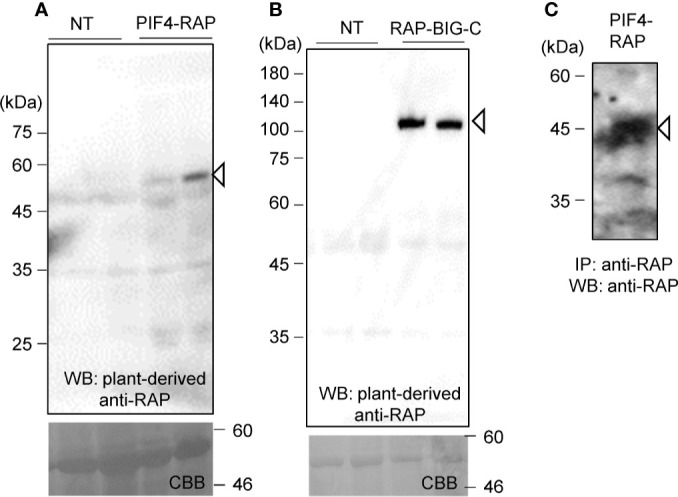
Applicability of the RAP tag in plant cells. PIF4-RAP **(A)** or RAP-BIG-C **(B)** were expressed in *N. benthamiana*. Total soluble extracts were separated by 12% SDS-PAGE and transferred onto PVDF membranes. PIF4-RAP and RAP-BIG-C were detected using PMab-2 purified from *N. benthamiana* leaves. **(C)** Purification of RAP-tagged PIF4 proteins from soluble extracts of *N. benthamiana* leaves. PIF4-RAP was purified using Dynabeads Protein G with plant-derived PMab-2.

The two genes were introduced into pBYR2HS. *Agrobacterium* harboring either pBYR2HS-dNPIF4RH or pBYR2HS-HRBIG12466 were then infiltrated into *N. benthamiana*. Soluble protein extracts were prepared from *N. benthamiana* leaves 4 days after incubation and separated by SDS-PAGE. PIF4-RAP and RAP-BIG-C proteins were detected using PMab-2 purified from *N. benthamiana* (**Figures 5A, B**).

Proteins were then purified using the RAP tag. The plant-derived PMab-2 antibody interacted with Dynabeads Protein G. Crude extracts from *N. benthamiana* infiltrated with pBYR2HS-dNPIF4RH were incubated with the PMab-2-bonded Dynabeads Protein G. The RAP-tagged target protein and PMab-2 complex were purified using Dynabeads Protein G. The immunoprecipitate was separated by SDS-PAGE and analyzed by immunoblotting with PMab-2 antibody. As shown in **Figure 5C**, the PIF4-RAP protein could be purified from crude plant extracts using the RAP tag and PMab-2.

**Figure 6:**
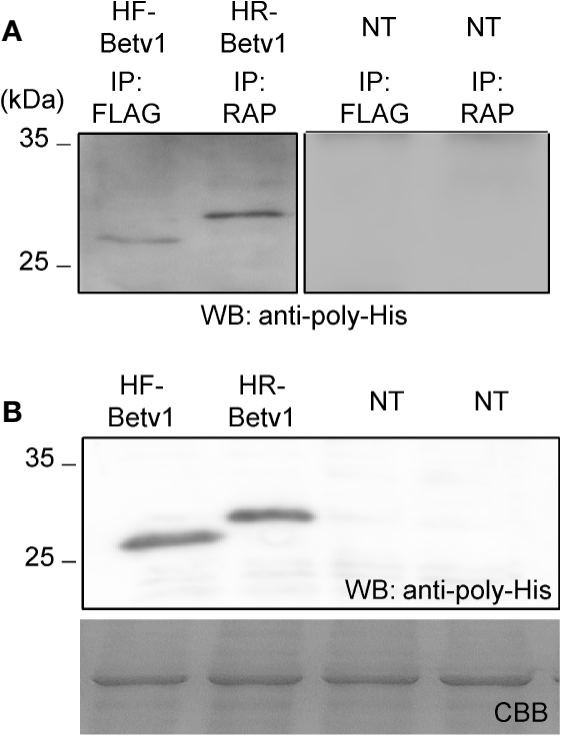
Comparison of the purification of RAP-tagged and FLAG-tagged Bet v 1. His-FLAG-Bet v 1 (HF-Betv1) and His-RAP-Bet v 1 (HR-Betv1) were expressed in *N. benthamiana* agroinfiltrated with pBYR2HS-HFBetv1Nt and pBYR2HS-HRBetv1Nt, respectively. **(A)** HF-Betv1 and HR-Betv1 were immunoprecipitated with anti-FLAG antibody (IP: FLAG) and PMab-2 (IP: RAP), respectively. *N. benthamiana* leaves (NT) were used as a negative control for immunoprecipitation. Immunoprecipitates were separated by SDS-PAGE, and western blot analysis was performed using anti-poly-His antibody to detect both HF-Betv1 and HR-Betv1. **(B)** Expression levels of HF-Betv1 and HR-Betv1 in *N. benthamiana* were confirmed by western blot analysis using anti-poly-His antibody. Agroinfiltrated leaves (100 mg) were harvested and mixed with the lysis buffer (500 μl). Soluble proteins were obtained by centrifugation. The same volume of the soluble protein mixture was separated by SDS-PAGE. Western blot analysis was performed using anti-poly-His antibody.

To assess if the RAP tagging system could be applied for detecting and purifying proteins, we compared its efficiency with that of the FLAG tagging system. We used Bet v 1 because His-FLAG-Bet v 1 (HF-Betv1) is known to be well-expressed in *N. benthamiana* (Yamada et al., 2020). His-RAP-Bet v 1 (HR-Betv1) was also expressed in *N. benthamiana*, and both HF-Betv1 and HR-Betv1 exhibited similar expression levels in *N. benthamiana* (**Figure 6B**). Then, the same amount of soluble proteins was used for immunoprecipitation with anti-FLAG antibody or plant-derived PMab-2, and western blot analysis was performed using anti-poly-His antibody (**Figure 6A**). Based on the resulting blot, the purification efficiency was found to be similar for both RAP and FLAG tagging systems.

## Discussion

An affinity tag system should possess both high affinity and specificity. PMab-2, a rat anti-mouse PDPN mAb, specifically recognizes the RAP tag epitope DMVNPGLEDRIE. In this study, high levels of PMab-2 production, reaching approximately 0.3 mg/gFW, were achieved in *N. benthamiana* using a newly developed expression system. The results confirmed that plant-produced PMab-2 retained its antigen-binding activity and specificity, similar to its mammalian CHO cell-derived counterpart (**Figure 4**). Furthermore, the efficiency of the RAP tag as a tool for protein detection in plant cells was demonstrated. RAP-tagged proteins expressed in *N. benthamiana* could be detected using plant-derived PMab-2 (**Figure 5**). In addition, the RAP tagging system was found to be as useful for protein purification as the FLAG tagging system (**Figure 6**).

Efficient complex formation is required to produce antibodies in plant cells. It has previously been shown that non-competing amplification of two different replicons can be achieved using a geminiviral replication vector (Phoolcharoen et al., 2011; Diamos et al., 2016; Rattanapisit et al., 2017). The most renowned and widely used transient expression system is the magnICON system (Marillonnet et al., 2005). In this system, viral vectors derived from the tobacco mosaic virus and potato virus X are used to produce recombinant proteins with two heterosubunits (Giritch et al., 2006). Secretory IgA, IgM, and heteromultimeric virus-like particles can be efficiently produced using these two viral vectors (Chen and Lai, 2013). Production of more than two heterosubunits is difficult because of the competing nature of many RNA viruses. Previously, four heterosubunits were successfully assembled using a non-replicating system, based on the ability of the cowpea mosaic virus to produce bluetongue virus-like particles (Thuenemann et al., 2013), albeit with a low recombinant protein yield.

In this study, the yield of PMab-2 in *N. benthamiana* was approximately 0.3 mg/gFW. Increasing the antibody yield would make it easier to obtain and purify antibodies from *N. benthamiana*. Changing the target location of recombinant proteins also affects their accumulation in *N. benthamiana*. Accumulation of HA was higher when the protein targeted the ER instead of the apoplast. The effect of leaf tissue necrosis caused by ER-targeted HA was suppressed at lower temperatures (Matsuda et al., 2017). On expressing the monoclonal antibody 14D9, which catalyzes enantioselective protonation (Zheng et al., 2004), fused with the vacuolar targeting signal (KISIA or NIFRGF) or ER retention signal (SEKDEK) in *N. benthamiana*, the accumulation of ER-mAb and vacuolar mAb was 10–15 folds higher than that of the secreted mAb (Ocampo et al., 2016). In the present study, PMab-2 was fused with KDEL. Another strategy is required to increase the antibody yield.

Small peptide tags are more useful than large peptide tags because the latter may perturb protein trafficking and function and protein-protein interactions (Lotze et al., 2016), and the former were developed to avoid potential interference with protein function. The His-tag is a well-known small tag consisting of a series of 6–12 histidines. This tag is used for purification by immobilized metal affinity chromatography (IMAC). IMAC is a cheap affinity chromatography technique when compared with affinity chromatography using an antibody. Although this is a clear advantage, IMAC captures other metal-binding proteins in addition to the His-tagged target proteins (Lichty et al., 2005). The His-tagging system with IMAC is effective for purifying large amounts of recombinant proteins. Conversely, other small tagging systems are useful for identifying protein functions. Several peptide tags, such as the FLAG (Xiao et al., 2019; Cui et al., 2020; Miura et al., 2020; Uemura et al., 2020), HA (Shinozawa et al., 2019; Kuhnert et al., 2020), and c-MYC (Earley et al., 2006; Baudisch et al., 2018; Roshan et al., 2018) tags are used in plants because of their specificity. These tags may affect the solubility or insolubility of the expressed proteins and their activities, depending on the characteristics of the expressed proteins (Ki and Pack, 2020). Thus, availability of different protein tagging systems is desirable.

In conclusion, the monoclonal antibody PMab-2 was expressed in *N. benthamiana*, and the RAP-tagged protein could be detected and purified using PMab-2. Plant-derived PMab-2 exhibited the same binding activity as the CHO-derived PMab-2. Furthermore, the RAP-tagged proteins were specifically recognized by PMab-2 in plant cells, allowing purification by affinity chromatography. These results indicate the potential of the RAP tag for use in plant cells and demonstrate that PMab-2 can be easily obtained using our transient expression system in *N. benthamiana*.

## Data Availability Statement

The datasets generated for this study are available on request to the corresponding author.

## Author Contributions

KM and YK conceived and designed the experiments. KM, HY, SN, and MK conducted the experiments and collected the data. KM and YK drafted and edited the manuscript.

## Funding

This work was supported by a Cooperative Research Grant #2020 of the Plant Transgenic Design Initiative (PTraD) by Gene Research Center, Tsukuba-Plant Innovation Research Center (T-PIRC), University of Tsukuba, JSPS Grant-in-Aid for Scientific Research on Innovative Areas (19H04637) to KM and AMED Grant Numbers JP19am0401013, JP19am0101078, and JP19ae0101028 to YK.

## Conflict of Interest

The authors declare that the research was conducted in the absence of any commercial or financial relationships that could be construed as a potential conflict of interest.
