# Supplementary material for: RAP Tag and PMab-2 Antibody: A Tagging System for Detecting and Purifying Proteins in Plant Cells
Source: Front Plant Sci. 2020 Sep 10;11:510444. doi: 10.3389/fpls.2020.510444 (PMC7511514; doi:10.3389/fpls.2020.510444)
Supplement: Supplementary file 1 [file DataSheet_1.pdf]

Table S1. Primers used for plasmid construction

Name of primer	DNA sequence (5'-to-3')
**pBYR2HS-PMabH**	
pBYR2HS-MYL-F	ACTGTTGATAGTCGATGTACTTA
pBYR2HS-KDEL-R	ATTCAGAATTGTCGACTTACAGCTC
**pBYR2HS-PMabL**	
pBYR2HS-MRF-F	ACTGTTGATAGTCGATGAGGTTC
pBYR2HS-stopC-R	ATTCAGAATTGTCGACTTAGCAC
**pBYR2HS-NHisRAP**	
pBYR2HS-Hisx6-F	ACTGTTGATAGTCGATGCATCATCACCACCATCAC
His-RAP-HRV3C	ATGCATCATCACCACCATCACGACATGGTGAATCCTGGTCTTGAGGATAGGATCGAGCTTGAGGTTTTGTTCCAGGGTCCT
pBYR2HS-HRV3C-R	ATTCAGAATTGTCGACAGGACCCTGGAACAAAACCTCAAG
**pBYR2HS-CRAPHis**	
pBYR2HS-HRV3C-F	ACTGTTGATAGTCGACCTTGAGGTTTTGTTCCAGGGTCC
HRV3C-RAP-His	CTTGAGGTTTTGTTCCAGGGTCCTGACATGGTGAATCCTGGTCTTGAGGATAGGATCGAGCATCATCACCACCATCACTAA
pBYR2HS-stopHis-R	ATTCAGAATTGTCGATTAGTGATGGTGGTGATGATG
**pBYR2HS-PIF4RH**	
pBYR2HS-dNPIF4-F	ACTGTTGATAGTCGATGTTTCTTGAAGATCAAGAAACTG
pBYR2HS-dNPIF4RH-R	AAACCTCAAGGTCGACGTGGTCCAAACGAGAACCGTCGGTG
**pBYR2HS-HRBIG12466**	
BIG-15297R	TCAATCTTTATCGATCGCCGATCTCAC
pBYR2HS-HR-BIG12466-F	CCAGGGTCCTGTCGACATGACAAAAAATCCATATTCGAGTG
pRI201-AtBIG-R	ATTCAGAATTGTCGACTCAATCTTTATCGATCGCCGATCTCAC
**pBYR2HS-HRBetv1Nt**	
HRV3C-Betv1Nt-F	CCAGGGTCCTGTCGACATGGGAGTTTTTAATTACGAAACTG
pRITetI-Betv1Nt-R	ATTCAGAATTGTCGACTTAATTGTATGCATCAGAGTGAGCG
